# Climate Change Effect on *Haematoxylum campechianum* and *Haematoxylum calakmulense* (Fabaceae): Are We Losing Our Natural Heritage in South‐Eastern Mexico?

**DOI:** 10.1002/ece3.72223

**Published:** 2025-10-22

**Authors:** Alexis Herminio Plasencia‐Vázquez, Anay Serrano‐Rodríguez, Annery Serrano Rodríguez, Yarelys Ferrer‐Sánchez

**Affiliations:** ^1^ Centro de Investigaciones Históricas y Sociales Universidad Autónoma de Campeche Campeche México; ^2^ Facultad de Ciencias Químico Biológicas Universidad Autónoma de Campeche Campeche México; ^3^ Facultad de Ciencias de la Ingeniería Universidad Técnica Estatal de Quevedo Quevedo Ecuador

**Keywords:** biodiversity conservation, climatic change, *Haematoxylum*, logwood, Maxent, niche modeling

## Abstract

Climate change threatens biodiversity and the balance of ecosystems. Ecological niche models (ENMs) permit exploring the consequences of climate change on species distribution patterns and are an applicable tool for the management of key species. *Haematoxylum campechianum* and *Haematoxylum calakmulense* are two plant species of economic and socio‐cultural interest for the Mesoamerican region. We aimed to characterize and identify the climatic niche overlap of both species and to assess the impact of climate change on their potential distribution in southeastern Mexico. We used 53 occurrence records of *H. calakmulense* and 604 of *H. campechianum* and a selection of climatic variables. After calibration and evaluation of the models, the best performing model for each species was selected. The models show a better performance with AUC values of 0.75 for *H. campechianum* and 0.66 for *H. calakmulense*. The niches of both species are similar, although not equivalent, but the variable with the greatest contribution in the case of *H. campechianum* is the mean annual temperature, whereas for *H. calakmulense* it is the mean temperature of the coldest quarter. It seems that *H. calakmulense* will lose more suitable areas in the future. In contrast, future projections for *H. campechianum* predict an area gain toward the southeast of Mexico, Belize, and Guatemala. This may indicate that *H. campechianum* is more resistant to climatic variation in the region, while *H. calakmulense* may have more problems with temperature variation soon. Our results should be considered in current and future reforestation plans to improve their efficiency.

## Graphical abstract

Climate change threatens biodiversity and the balance of ecosystems, specifically in key species. It seems that *Haematoxylum calakmulense* will lose more suitable areas than *Haematoxylum campehianum* in the future. Our results should be considered in current and future reforestation plans to improve their efficiency.

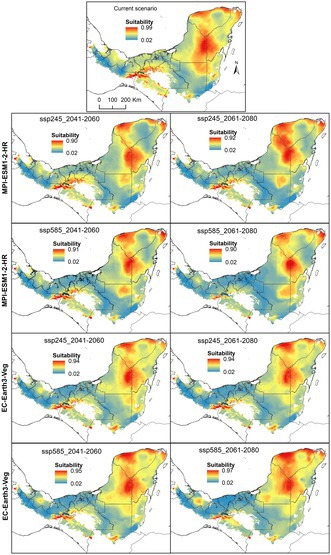

## 1 Introduction

The distribution of plant species is determined by many factors, including historical elements and their ecological adaptations such as dispersal strategies (de Freitas Alves and de Santana 2022). Relationships between species also influence the geographical location of their populations (Wisz et al. 2013). For example, this can be due to the displacement or prevalence of a species caused by allelopathy (Schandry and Becker 2020) or the presence of pathogens, facilitation or exclusion by soil‐modifying species (Ma et al. 2017), or competition (Capitán et al. 2021). In addition to biotic factors, the influence of surrounding environmental conditions is crucial for the presence of species in each ecosystem. For example, climatic factors influence the functional traits of plant leaves by controlling plant metabolism, growth, and developmental processes (Wang et al. 2022). These abiotic factors, which encompass climatic conditions, can vary both spatially and temporally, leading to changes in species distribution patterns in both directions.

Climate change is one of the most worrying phenomena of recent decades because it threatens biodiversity and the balance of ecosystems (Bellard et al. 2012). In the face of relatively rapid climate change, plant species are unable to adapt and respond to the variations that occur in a short period of time (Davis and Shaw 2001). This leads to a decrease in the survival of individuals of different plant species, resulting in habitat reduction and fragmentation (Wiens 2016) and impacts on ecosystem services to society (Weiskopf et al. 2020). Climate change may increase the likelihood of some species expanding their populations, including invasive alien species that compete with native species (Yuan et al. 2021). Conversely, many native or endemic species may see their ranges shrink or even disappear in response to these changes (Manes et al. 2021).

Ecological niche models are a useful tool for exploring these relationships and predicting the effects of climate change under different current and future scenarios (Peterson et al. 2012). These models use species occurrence as a function of specific environmental variables (Elith and Leathwick 2009). Understanding the relationships between species and climate is crucial for anticipating the effects of climate change and making decisions to mitigate its impacts (Peterson et al. 2003). Research into these predictions is particularly urgent for key species of economic and/or heritage value. Some plant species may be more vulnerable to climate change; for example, those with narrow tolerance ranges or environmental niches (Hanski et al. 1993). On the other hand, lowland species may also be affected by sea‐level rise (Loarie et al. 2009). There are currently many statistical tools available for predicting species distribution. These modeling methods vary in complexity and may include machine learning algorithms based on regression and trees such as Generalized Linear Models (GLM), Generalized Additive Models (GAM), Random Forest (RF), Boosted Regression Trees (BRT), Bayesian Additive Regression Trees (BART), and Maximum Entropy (MaxEnt). However, MaxEnt is a reliable option for working with scarce or biased presence‐only data, particularly in studies of rare or endemic species (Qazi et al. 2022). There are also tools available to help implement MaxEnt, which facilitate model selection through appropriate calibration (complexity adjustment), and its models often demonstrate high predictive accuracy and stability (Duan et al. 2014; Warren and Seifert 2011).

Two species of the genus *Haematoxylum* occur in southeastern Mexico in association with flooded lowlands. *Haematoxylum campechianum* L. can be found in different plant formations as isolated individuals or in groups of several individuals forming “tintales” (Niembro 2002). This species has a history of exploitation for obtaining natural dyes, being the main resource in the region during colonial times (Villegas and Torras 2014) and is therefore considered a species with heritage value. Its distribution seems to overlap with that of *Haematoxylum calakmulense*, the morphologically most similar sister species (Cruz Durán and Sousa 2014). However, *H. calakmulense* has been reported to be found toward the center of the Yucatán Peninsula, while the distribution of *H. campechianum* extends to the coast reaching the states of Tabasco and Veracruz in the north (Cruz Durán and Sousa 2014; Chablé‐Vega et al. 2019; Serrano‐Rodríguez et al. 2024). In addition, we can find *H. campechianum* naturalized in the Caribbean Islands (Adams 1972; García‐Beltrán et al. 2024) and introduced and cultivated in other countries in distant continents, such as Australia and Africa (Gurib‐Fakim 2005; GBIF 2024a). Both species have melliferous potential, especially *H. campechianum* , which is listed as an important species for meliponiculture (Ríos‐Oviedo et al. 2024) and beekeeping (Cetzal‐Ix et al. 2019; Coh‐Martínez et al. 2019), which is one of the most important economic activities in the region (Villanueva and Colli‐Ucan 1996).

Knowing the relationship between the current and future distribution patterns of both *Haematoxylum* species and their relationship with climate would allow us to identify priority areas and improve the efficiency of reforestation plans with native species. Due to the importance of these species and their potential vulnerability to climate change, this work aims to (1) characterize the climatic niche of *H. campechianum* and *H. calakmulense* and identify overlaps, (2) assess the impact of climate change on the potential distribution of both species present in south‐eastern Mexico using ecological niche models.

## 2 Materials and Methods

### 2.1 Ocurrence Records

A total of 143 occurrence records of *H. calakmulense* and 3150 of *H. campechianum* were collected from the Global Biodiversity Information Facility database (GBIF 2024a, 2024b), herbarium specimens, and fieldwork from previous studies (Plasencia‐Vázquez et al. 2017, 2020; Chablé‐Vega et al. 2019; García‐González et al. 2021). Duplicate records, datasets with insufficient data or doubtful locations, and occurrences closest to each other at a distance of ~1 km were eliminated. For *H. campechianum* , records outside Mexico and the Caribbean islands, where it is likely to have been cultivated, were removed. Modeling within a region reachable by dispersal on a relevant timescale is important because it reduces biases in prevalence and environmental availability (Barve et al. 2011). Furthermore, the data must be thoroughly cleaned because misidentification can unduly shift or expand the predicted distribution toward the niche of the occurrence conditions of the confusing data and alter the evaluation values of the final models. Conversely, records of cultivated individuals introduce environmental conditions that are not representative of the natural niche (Coca‐de‐la‐Iglesia et al. 2023). The R spThin library package (Aiello‐Lammens et al. 2015) was used for database cleaning. Finally, 53 records of *H. calakmulense* and 604 records of *H. campechianum* were used. The training of the models was based on 85% of the data for each species, with a further 15% of the data reserved for evaluation.

### 2.2 Environmental Data Selection. Calibration and Evaluation Models

We used 15 climate variables available in the WorldClim version 2.1 database (http://worldclim.org) (Fick and Hijmans 2017), with a resolution of 30 arc‐sec (approximately 1 km). The variables bio8 (Mean Temperature of Wettest Quarter), bio9 (Mean Temperature of Driest Quarter), bio18 (Precipitation of Warmest Quarter), and bio19 (Precipitation of Coldest Quarter) were discarded since they are considered statistical artifacts due to their redundant nature or derived from the monthly variables (which causes collinearity between them), which have been eliminated in other studies with similar protocols (Alkishe et al. 2017; Datta et al. 2020). In addition, discontinuities related to sudden changes in the quarterly periods used to calculate these variables have been identified (Booth 2022). To discard variables and avoid collinearity between them, four groups of five or six variables were selected using different methods. To select the variables in the first group, we used the correlation between pairs of variables, eliminating those with a correlation coefficient greater than 0.7. The second group was selected using the variance inflation factor (VIF), a useful tool for detecting multicollinearity (Oksanen et al. 2017). The third and fourth groups of variables were selected using principal components analysis, along with a combination of the variables that contributed most to the multivariate model and those identified in the literature as potentially important for the study species (Plasencia‐Vázquez et al. 2017; Serrano‐Rodríguez et al. 2024).

### 2.3 Calibration, Evaluation and Selection of Ecological Niche Models

Ecological niche models were calibrated, evaluated, and selected using the MaxEnt algorithm (Phillips et al. 2006), through the kuenm library for R (Cobos et al. 2019). A buffer area of 50 km around the selected occurrence points was used to calibrate the models. A total of 504 models were evaluated, considering 18 configurations of regularization multipliers, 7 combinations of entity classes, and 4 different sets of environmental variables. Model performance was evaluated based on statistical significance (partial ROC), omission rates (OR), and the Akaike information criterion corrected for small sample size (AICc). The final models were built by projecting over the area of interest corresponding to ecoregions containing at least one point occurrence in southeastern Mexico, Guatemala, and Belize. To analyze the potential impact of climate change in the future, they were projected over two time periods (2041–2060 and 2061–2080), considering two scenarios of change (ssp245 and ssp585) for two different circulation models, MPI‐ESM1‐2‐HR and EC‐Earth3‐Veg, which present good resolution and good performance for the region (Almazroui et al. 2021). These two circulation models perform well in extreme climates, including those involving extreme precipitation and temperature, such as the droughts and high temperatures experienced in Latin America and the Caribbean (Avila‐Diaz et al. 2023). Predictions were obtained using clamped extrapolation, ensuring that only a known range of values for the species was considered to avoid unrealistic overestimates. Finally, the binary maps were obtained using the ‘10th percentile training presence’ as the cut‐off threshold.

### 2.4 Niche Overlap

To characterize the environment occupied by the species and explore the environmental conditions available in the accessible area, a principal component analysis (PCA) was performed. The density of occurrence of both species in the environmental space was plotted with the first two components on a 100 × 100 grid (Broennimann et al. 2012). This plot makes it possible to visualize the extent and location of the niche in the environmental space in relation to the environmental conditions available in the study area, using kernel smoothers. This was done using the ecospat package in R (Broennimann et al. 2012; Petitpierre et al. 2012).

Niche overlap between the two species was calculated using Schoener's *D* statistic (Warren et al. 2008). The value of *D* ranges from 0, when two species have no overlap in environmental space, to 1 when two species share the same environmental space. The niche equivalence test allowed us to assess whether the ecological niches were significantly different from each other and whether the two niche spaces were interchangeable. If the niche overlap value of the compared species was significantly lower than the null distribution overlap values (*p* < 0.05), nonequivalence between the niches was assumed. A niche similarity test was also performed, which assesses whether ecological niches are more different than expected by chance, considering the environmental variability of the surrounding environment (Warren et al. 2010).

## 3 Results

### 3.1 Climatic Niche Models

The models obtained show a better performance than expected by chance, with acceptable fits AUC values of 0.75 for *H. campechianum* and 0.66 for *H. calakmulense*. Furthermore, the models presented acceptable evaluation parameters, with a small number of parameters, and the partial ROC bootstrap tests showed significant values, with *p* < 0.001 in both cases and dropout rates less than 0.1 (Table 1). The final model selected for *H. campechianum* (Table 1) was constructed with the first set of proposed variables, including bio1 (Annual Mean Temperature), bio2 (Mean Diurnal Range), bio3 (Isothermality), bio12 (Annual Precipitation), and bio15 (Precipitation Seasonality). Bio1 was the variable with the highest contribution, accounting for more than 70% (Table 2), and to a lesser extent bio2. For *H. calakmulense*, the variables included in the final model were bio2, bio3, bio11 (Mean Temperature of Coldest Quarter), bio15, bio16 (Precipitation of Wettest Quarter), and bio17 (Precipitation of Driest Quarter). All variables included in this model had a contribution greater than 10% (Table 2).

**TABLE 1:** Selected ecological niche models (in bold) for *Haematoxylum campechianum* and *Haematoxylum calakmulense* using the maximum entropy algorithm (MaxEnt).

Species	rm	Feature	Variables	Mean AUC ratio	pval pROC	Omission rate at 10%	AICc	Delta AICc	W AICc	Number of parameters
*H. campechianum*	**0.2**	**lqp**	**Set1**	**1.271**	**0**	**0.096**	**14,755.371**	**0.000**	**0.933**	**14**
*H. calakmulense*	**0.4**	**p**	**Set4**	**1.174**	**0**	**0.000**	**1210.387**	**0.000**	**0.086**	**4**
	0.5	p	Set4	1.177	0	0.000	1210.541	0.154	0.079	4
	0.6	p	Set4	1.164	0	0.000	1210.727	0.340	0.072	4
	0.7	p	Set4	1.175	0	0.000	1210.946	0.560	0.065	4

*Note:* Rm (regularization multiplier); Features l (linear), p (product), q (quadratic); AICc (Akaike information criterion corrected); Delta AICc (Delta AICc); W AICc (weight Akaike information criterion corrected). Set1: bio1 (Annual Mean Temperature), bio2 (Mean Diurnal Range), bio3 (Isothermality), bio12 (Annual Precipitation) and bio15 (Precipitation Seasonality). Set 4: bio2, bio3, bio11 (Mean Temperature of Coldest Quarter), bio15, bio16 (Precipitation of Wettest Quarter) and bio17 (Precipitation of Driest Quarter). The model highlighted in bold was the selected model.

**TABLE 2:** Contribution of variables in the final climatic niche models constructed for *Haematoxylum calakmulense* and *Haematoxylum campechianum* for the ecoregions of Mexico and Central America where they are distributed.

ID	Variable	% contribution	
		*H. calakmulense*	*H. campechianum*
bio1	Annual mean temperature	—	73.71
bio2	Mean diurnal range	10.67	14.37
bio3	Isothermality	12.26	1.52
bio11	Mean temperature of coldest quarter	22.77	—
bio12	Annual precipitation	—	6.64
bio15	Precipitation seasonality	19.59	3.76
bio16	Precipitation of wettest quarter	18.23	—
bio17	Precipitation of driest quarter	16.48	—

**FIGURE 1:**
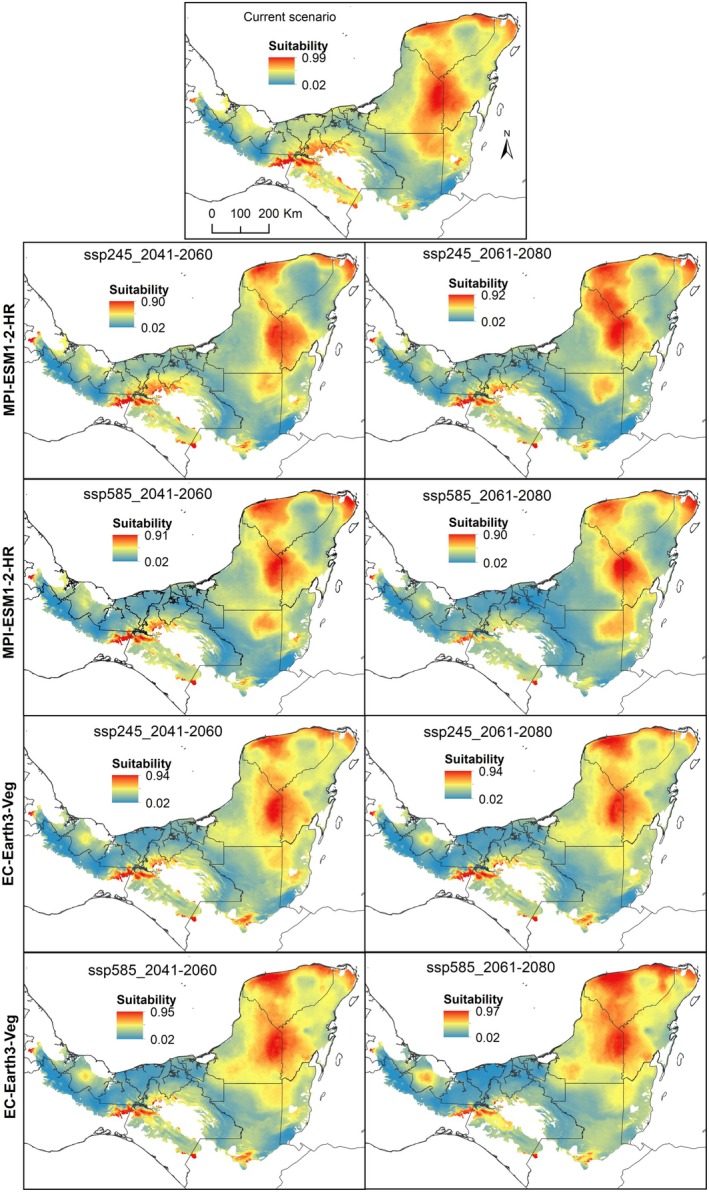
Suitability according to climate niche models obtained for *Haematoxylum calakmulense*, projected in the ecoregions of Mexico and Central America where at least one occurrence record is found, for the present and for two future time periods (2041–2060 and 2061–2080), considering two scenarios of moderate (ssp245) and extreme (ssp585) change for two different circulation models, MPI‐ESM1‐2‐HR and EC‐Earth3‐Veg.

**FIGURE 2:**
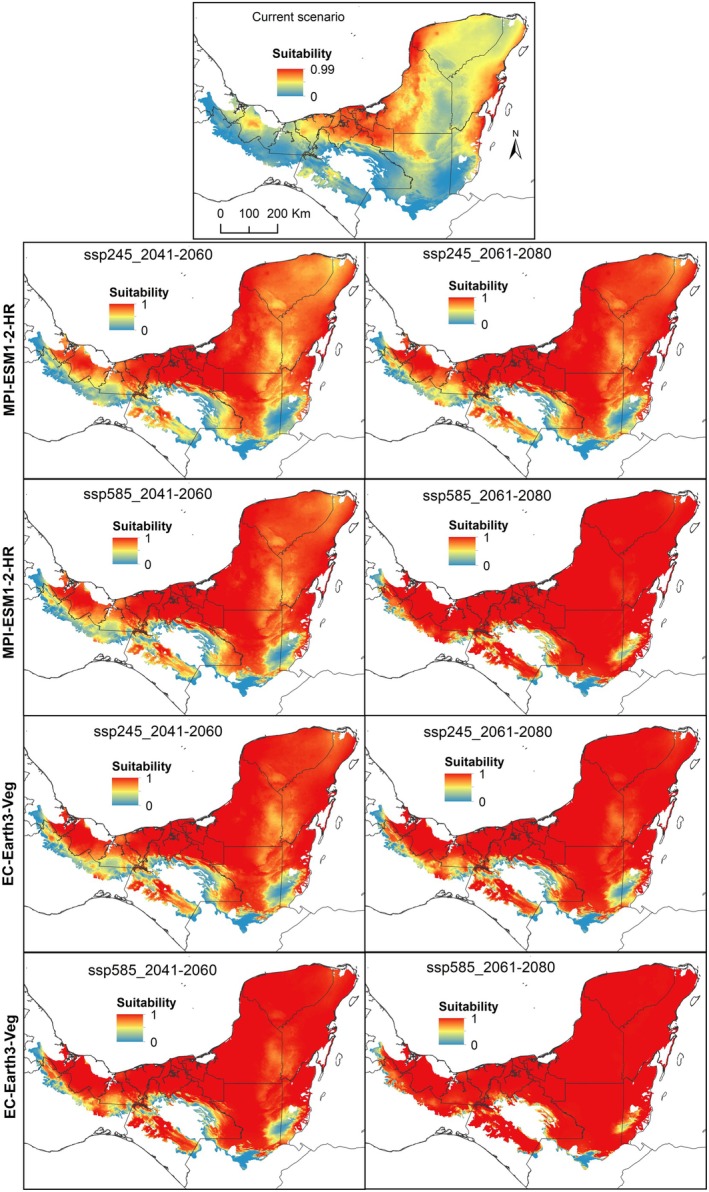
Suitability according to climate niche models obtained for *Haematoxylum campechianum* , projected in the ecoregions of Mexico and Central America where at least one occurrence record was found for the present and two future time periods (2041–2060 and 2061–2080), considering two scenarios of change (ssp245 and ssp585) for two different circulation models, MPI‐ESM1‐2‐HR and EC‐Earth3‐Veg.

**FIGURE A1:**
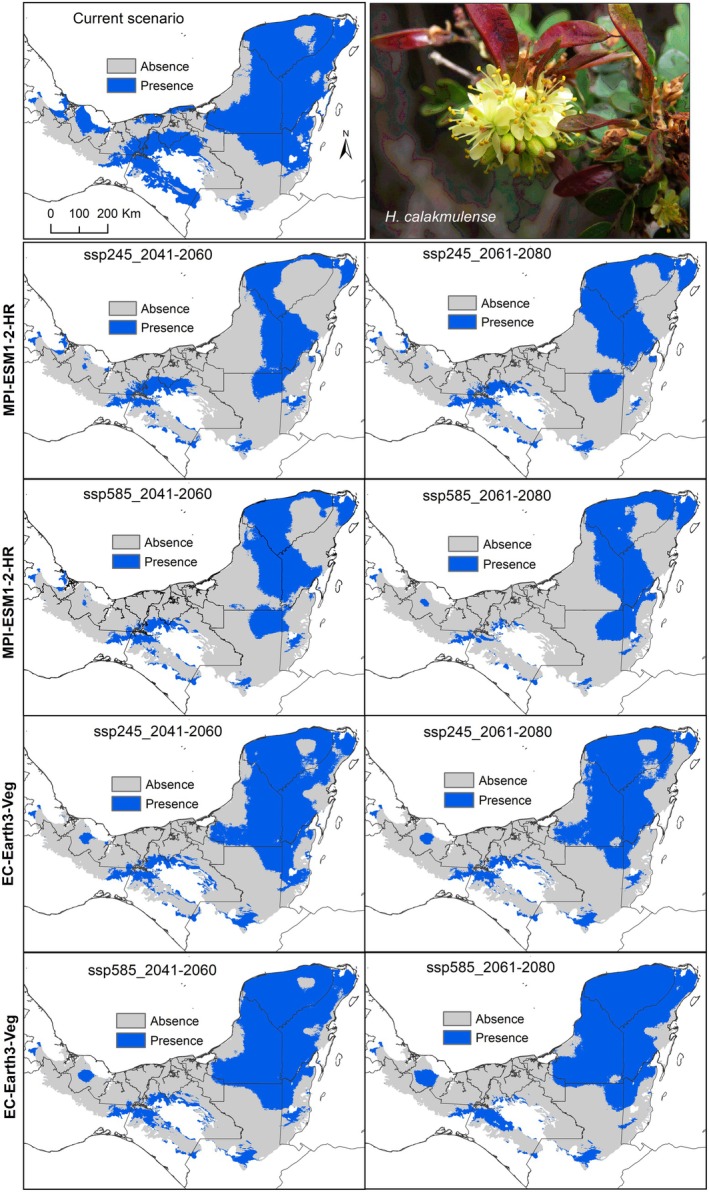
Location of suitable areas according to climate niche models, obtained for *Haematoxylum calakmulense*, projected in the ecoregions of Mexico and Central America where at least one occurrence record is found for the present and in two future time periods (2041–2060 and 2061–2080) considering two scenarios of change (ssp245 and ssp585) for two different circulation models, MPI‐ESM1‐2‐HR and EC‐Earth3‐Veg.

**FIGURE A2:**
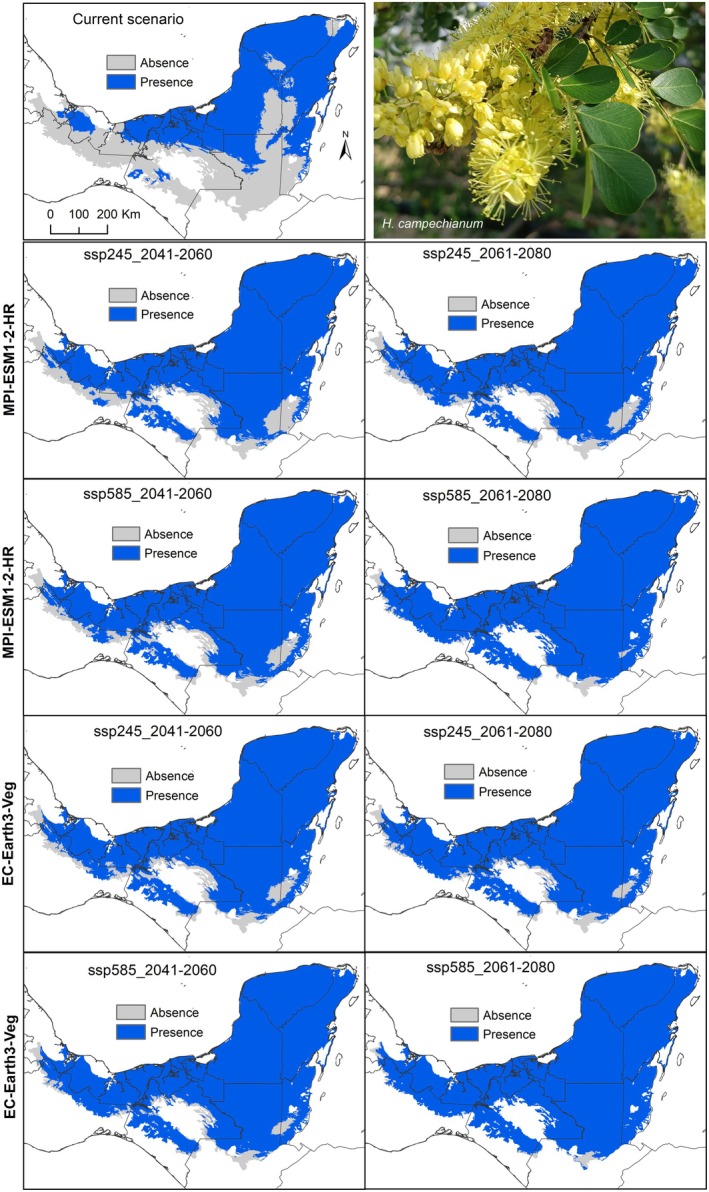
Location of suitable areas according to climate niche models, obtained for *Haematoxylum campechianum* , projected in the ecoregions of Mexico and Central America where at least one occurrence record is found for the present and in two future time periods (2041–2060 and 2061–2080) taking into account two scenarios of change (ssp245 and ssp585) for two different circulation models, MPI‐ESM1‐2‐HR and EC‐Earth3‐Veg.

In the current scenario, *H. campechianum* has a potential geographic distribution of approximately 1.77 × 10^5^ ha, while the potential suitable area for *H. calakmulense* covers 1.89 × 10^7^ ha, being larger for the latter species (Figures A1 and A2; Table A1). The highest suitability values for *H. calakmulense* are found in the center of the Yucatan Peninsula, on the border between the states of Campeche and Quintana Roo, but also on the northern coast of Yucatan State (Figure 1) and in Chiapas, where there are no records of the species to date. On the other hand, the most suitable areas for *H. campechianum* are in the west of the Yucatan Peninsula toward the coast of the states of Campeche and Tabasco, but also on the south coast of the state of Quintana Roo and the north coast of Belize (Figure 2).

**FIGURE 3:**
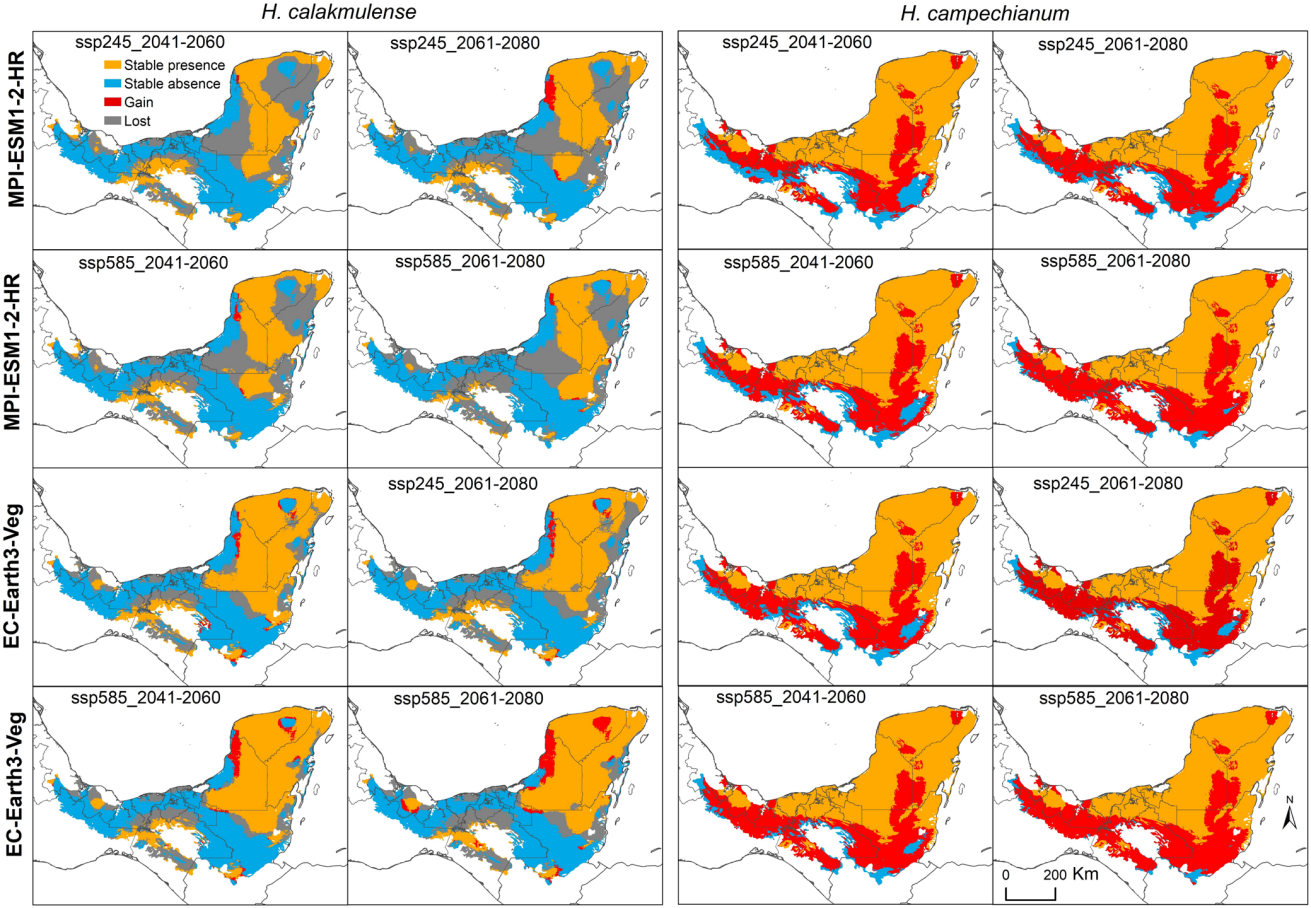
Areas of stable potential distribution (orange), stability of areas of absence (blue), potential area loss in the future (gray) and potential area gain (red) according to climate niche models, projected for *Haematoxylum calakmulense* and *Haematoxylum campechianum* , projected in the ecoregions of Mexico and Central America where there is at least one occurrence record for the present and for two future periods (2041–2060 and 2061–2080), considering two scenarios of change (ssp245 and ssp585) for two different circulation models, MPI‐ESM1‐2‐HR and EC‐Earth3‐Veg.

**FIGURE 4:**
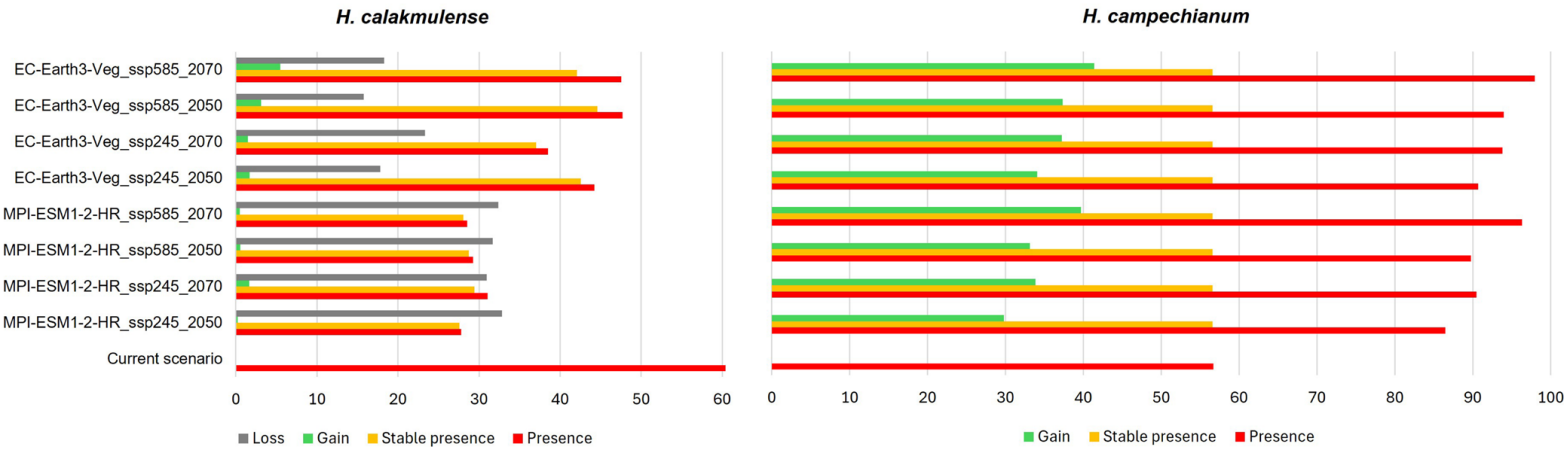
Percentage of suitable area for *Haematoxylum calakmulense* and *Haematoxylum campechianum* in relation to the total area (red). The percentage of suitable area that remain stable over time (orange), the gain (green), and loss (gray) of suitable area in two future time periods (2041–2060 and 2061–2080) considering two scenarios of change (ssp245 and ssp585) for two different circulation models, MPI‐ESM1‐2‐HR and EC‐Earth3‐Veg, are shown.

Environmental suitability in the *H. calakmulense* models for the future (Figure 1) shows a decrease in the potential area for the two time periods (2041–2060 and 2061–2080), in the moderate (ssp245) and extreme (ssp585) change scenarios and in the two circulation models studied (MPI‐ESM1‐2‐HR and EC‐Earth3‐Veg). In contrast, future models predict an increase in the area potentially suitable for *H. campechianum* (Figure 2). In the states of Campeche and Quintana Roo in Mexico, *H. calakmulense* appears to lose more suitable area in the future (Figure 3). In contrast, future projections for *H. campechianum* do not predict a loss of suitable area, but rather an increase toward the center of the Yucatan Peninsula, Tabasco, Chiapas, Belize, and Guatemala. The largest loss of suitable area for *H. calakmulense* is recorded for the MPI‐ESM1‐2‐HR circulation model between 2041 and 2060 in a moderate scenario, corresponding to a loss of 8,620,319.46 ha (Figure 4). This circulation model predicts greater losses of suitable habitat for *H. calakmulense* in southern Campeche and around the border between the states of Yucatán and Quintana Roo. However, there do not appear to be drastic differences between time periods or between moderate and extreme change scenarios.

### 3.2 Niche Breadth and Overlap

**FIGURE 5:**
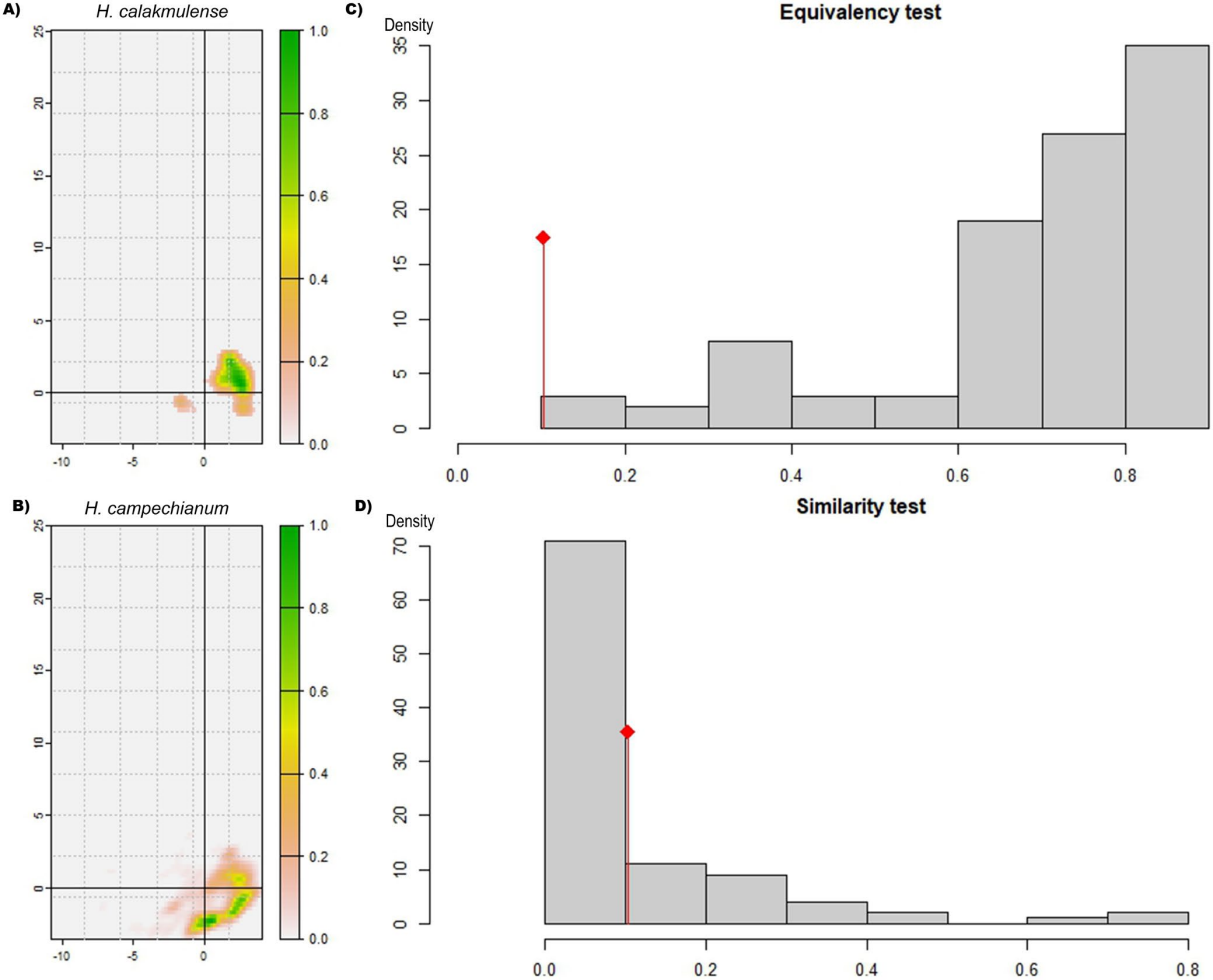
Density of occurrence in the environmental space of *Haematoxylum calakmulense* (A) and *Haematoxylum campechianum* (B) using the first two components of a principal component analysis. Results of the test of equivalence (*p* > 0.05 assumes no niche equivalence) and similarity (*p* > 0.05 indicates similarity) between the climatic niches of *H. calakmulense* (C) and *H. campechianum* (D).

Principal component analysis to characterize the climatic niche showed that both species have a relatively marginal position with respect to the available environmental conditions (Figure 5A,B). The first component explains 41.89% of the variability, while the second component contributes 28.25%. The environmental niches of both species are located in the same region of the graph (Figure 5A,B): below 3 on the first component (*y*‐axis) and above −0.3 on the second component (*x*‐axis). However, the presence densities of the two species are somewhat segregated in environmental space. The highest presence densities for *H. calakmulense* are above zero in the first component, while *H. campechianum* is found below zero in the presence density areas of environmental niches. The test of equivalence and similarity between the niches showed that they are not equivalent but similar (Figure 5C,D).

## 4 Discussion

### 4.1 Climatic Niche Models

Our results show the potential distribution of *H. calakmulense* and *H. campechianum* considering ecological niche models, which allowed us to predict the effects of climate change in the future under different scenarios for two species of economic and cultural value (Campos García and Leyva Morales 2023). This study demonstrates that climate change will favor the potential expansion of *H. campechianum* , in contrast to the projected reduction in habitat for *H. calakmulense*. This is a key finding that will inform conservation actions.

The areas where the environmental suitability of *H. campechianum* is greatest coincide with those reported by Serrano‐Rodríguez et al. (2024), although our maps were made using only climatic variables, excluding other variables such as soil characteristics. This suggests that for some species, such as *H. campechianum* , climatic variables are currently sufficient to obtain relatively reliable models (Aguirre‐Gutiérrez et al. 2015). In the case of *H. calakmulense*, it may be useful to include other variables to improve the performance of current models. However, predicting the effects of climate change requires projecting these variables into the future, which is difficult for non‐climatic variables. Some studies assume that topography and abiotic soil variables will not change in the future, but this may introduce significant bias in a region undergoing constant land use change (Ellis et al. 2017).

The variable that contributed most to model performance for *H. campechianum* was mean annual temperature (bio1), with the warmest areas being the most suitable for the species, at least within the known range of values. Climatic variation can significantly affect leaf nutrient content (Zhang et al. 2020; Wang et al. 2022). Low temperatures cause a variation in leaf nitrogen content and thus a change in the photosynthetic process (Borer et al. 2013). On the other hand, as temperature increases, soil microbial respiration is enhanced, the efficiency of mineralization and decomposition of organic matter increases, and the effective nitrogen and phosphorus content of the soil increases (Mondal et al. 2016), available for plant growth.

The suitability of *H. calakmulense*, on the other hand, appears to be more related to precipitation or a combination of precipitation and temperature extremes. Temperature and precipitation factors together determine nutrient changes in plant leaves (Fang et al. 2021) and alter soil nutrient availability. As precipitation declines and soils experience drought stress, plants may respond to water stress by altering functional traits to some extent (Picotte et al. 2009). The Yucatan Peninsula hosts increasingly unpredictable environments that suffer from extreme drought (Márdero et al. 2012), which may be of concern for the survival of species that depend on precipitation during at least one season of the year, such as *H. calakmulense*.

Increasing temperatures and precipitation patterns will also impact the incidence of pests and diseases. For example, increased pressure from coffee rust (*Hemileia vastatrix*) is predicted in Mexico (Donatti et al. 2019). Generally, rising minimum and maximum temperatures reduce the thermal comfort of many species, causing them to shift their ranges primarily to higher latitudes or altitudes (Andrade‐Velázquez et al. 2021). Furthermore, these variations in maximum and minimum temperatures generally reduce the thermal comfort of many species, which tend to shift their ranges primarily to higher latitudes or altitudes (Sampayo‐Maldonado et al. 2022; Aguirre‐Gutiérrez et al. 2025). This can reduce the area with favorable conditions for species and therefore increase competition (Ramírez‐Barahona et al. 2023). For example, extra heat can increase the growth and reproduction of mangroves and expand their distribution limits, although other factors such as salinity, sea level, and storms also play a role (Ruiz 2024).

The resulting models of potential future distribution predict a reduction in the suitable area for *H. calakmulense*. The vulnerability of this quasi‐endemic species in the region is compounded by the possibility that rapid climate change will not allow many species to develop the adaptations necessary to survive (Davis and Shaw 2001; Etterson and Shaw 2001). In addition, certain species may require much faster dispersal rates than those observed in previous seasons (Malcolm et al. 2002) and may not be able to keep pace with rapid climate change.

Projected meteorological data indicate an increase in the frequency and duration of droughts in the hydrological regions of central and southern Mexico (Velázquez‐Zapata and Dávila‐Ortiz 2025). These findings emphasize the importance of improving water management policies and adaptation strategies to mitigate the anticipated effects of climate change in Mexico. Meanwhile, seasonal flooding acts as an abiotic filter for the distribution of plant species in the Yucatán Peninsula (Mendoza‐Arroyo et al. 2024). Changes in future precipitation could alter the typical seasonality of the region's flooded forests, potentially affecting *Haematoxylum* species on the Yucatán Peninsula differently. Although the distribution model of *H. calakmlense* identified in this study is relatively broad, its individuals are less abundant than those of *H. campechianum* , which often dominates, forming “tintales.” It is possible that other biotic and abiotic factors not included here determine the differences in the distribution patterns of individuals and populations of both species. *Haematoxylum campechianum* appears to have greater plasticity and resistance to extreme ecological conditions than *H. calakmulense*. Other species traits, such as niche position, may also influence range size, but a systematic review of the current evidence is required (Slatyer et al. 2013).

It is important to note that our models did not consider the impact of sea level variation, or land use changes caused by human activity, on the distribution patterns of the species under study. However, we believe this is an important factor to consider in the discussion, particularly about populations of *H. campechianum* living near the coast, which are greatly impacted by the complex hydrological processes of these seasonally inundated ecosystems. Significant variations in the chemical composition of the water have been observed in recent decades (Narvaez‐Montoya et al. 2025). The main factors triggering high salinity levels in groundwater are the mixing of seawater in the north of the peninsula, the dissolution of carbonates and gypsum in the interior and south, and the presence of contaminants. A projected sea level rise of 20 cm on the peninsula would result in seawater intruding hundreds of meters, or even kilometers, inland. This would reduce the thickness of the usable aquifer (freshwater) and increase the vulnerability of ecosystems that depend on the water cycle (Sánchez‐García et al. 2023). Furthermore, climate change may alter hydrogeological patterns in the karst environment, affecting the dissolution rate of carbonates and gypsum. The distribution of *H. campechianum* and *H. calakmulense* may also be affected by an increase in land use for agriculture, livestock farming, and real estate development in the future (Ramirez‐Deldado et al. 2014; Bonilla‐Moheno et al. 2021).

In addition, we must be cautious in interpreting models, as other factors influence the future distribution of species. For example, landscape fragmentation and the dispersal abilities of individual species cause climate niche model assumptions to be inaccurate (Wang et al. 2022). Failure to account for these factors will lead to under‐ or overestimation of future species distributions, resulting in high uncertainty in the results (Wang et al. 2022). Factors determining germination should also be considered for *H. campechianum* , as it is tentatively considered to be a recalcitrant species due to a high percentage of germination in seeds in the first year and an accelerated aging process with low viability and germination in seeds stored for more than 1 year (Euan‐Tun et al. 2021).

### 4.2 Implications for Conservation

Our results suggest that we should be cautious when planning reforestation with *H. campechianum* , which appears to be more resilient to climate change. We consider it a useful species for afforestation plans, as its resilience will ensure its survival, but it should only be carried out in areas of historical and potential distribution. In the case of rare plants, they are often weak competitors that have found refuge in hostile environments where they may be locally abundant (Boulangeat et al. 2012). This may be the case for *H. calakmulense*, but corroboration with fieldwork is needed to describe the emergent properties of its populations. Compared to widely distributed species, they are expected to be more vulnerable to environmental change due to a restricted niche and probably other factors such as reduced dispersal ability (Holt et al. 2004; Baumberger et al. 2021). Knowing the vulnerability factors of rare plant species is challenging for conservationists trying to manage their natural populations for conservation (Scheele et al. 2019; Pogorzelec et al. 2020). Furthermore, knowing these relationships with the environment allows the ecosystem services of each species to be valued and the resources they provide to be used in a sustainable manner (Pandey 2015).

## Author Contributions

**Alexis Herminio Plasencia‐Vázquez:** conceptualization (equal), data curation (equal), formal analysis (equal), investigation (equal), methodology (equal), software (equal), supervision (equal), validation (equal), visualization (equal), writing – review and editing (equal). **Anay Serrano‐Rodríguez:** conceptualization (equal), data curation (equal), formal analysis (equal), investigation (equal), methodology (equal), software (equal), validation (equal), visualization (equal), writing – original draft (equal), writing – review and editing (equal). **Annery Serrano Rodríguez:** conceptualization (equal), data curation (equal), formal analysis (equal), investigation (equal), methodology (equal), supervision (equal), visualization (equal), writing – review and editing (equal). **Yarelys Ferrer‐Sánchez:** conceptualization (equal), data curation (equal), formal analysis (equal), investigation (equal), methodology (equal), visualization (equal), writing – review and editing (equal).

## Conflicts of Interest

The authors declare no conflicts of interest.

## Data Availability

Species occurrence data are available in the free repository: https://doi.org/10.5281/zenodo.15468259.

## Appendix A

**TABLE A1:** Area of presence, absence in binary maps, as well as areas of stability (presence and absence), loss and gain of suitable habitat in future projections.

Current scenario	Presence	Absence	Stable presence	Stable absence	Gain	Loss
*Haematoxylum calakmulense*						
	18,907,700.6	12,399,065.8				
MPI‐ESM1‐2‐HR_ssp245_2041–2060	8,704,023.7	22,599,084.9	8,620,319.5	12,313,159.5	74,572.7	10,265,513.0
MPI‐ESM1‐2‐HR_ssp245_2061–2080	9,726,735.9	21,577,678.0	9,206,223.6	11,874,956.1	510,994.8	9,680,626.3
MPI‐ESM1‐2‐HR_ssp585_2041–2060	916,1901.8	22,143,660.8	8,985,334.8	12,221,177.4	166,967.1	9,900,997.1
MPI‐ESM1‐2‐HR_ssp585_2061–2080	8,930,362.3	2,2376,867.2	8,767,705.0	12,232,504.5	156,794.9	10,120,921.2
EC‐Earth3‐Veg_ssp245_2041–2060	13,847,877.7	17,457,076.8	13,312,840.1	11,862,576.7	523,666.0	5,575,812.1
EC‐Earth3‐Veg_ssp245_2061–2080	12,049,547.8	19,254,937.0	11,585,069.4	11,931,198.9	456,856.6	7,304,322.8
EC‐Earth3‐Veg_ssp585_2041–2060	14,930,217.5	16,374,827.9	13,949,580.4	11,418,869.4	968,750.4	4,938,787.2
EC‐Earth3‐Veg_ssp585_2061–2080	14,889,329.7	16,417,243.8	13,164,674.8	10,671,957.3	1,715,911.0	5,725,173.3
*Haematoxylum campechianum*						
	17,703,553.0	13,517,833.9				
MPI‐ESM1‐2‐HR_ssp245_2041–2060	26,990,210.0	4,224,240.3	17,704,658.3	4,254,185.2	9,333,276.9	0.0
MPI‐ESM1‐2‐HR_ssp245_2061–2080	28,234,880.3	2,975,004.1	17,704,654.8	2,991,627.6	10,593,739.7	0.0
MPI‐ESM1‐2‐HR_ssp585_2041–2060	28,027,461.1	3,186,721.5	17,704,653.0	3,206,847.7	10,378,036.9	0.0
MPI‐ESM1‐2‐HR_ssp585_2061–2080	30,062,946.6	1,150,788.0	17,704,713.1	1,149,607.0	12,426,316.7	0.0
EC‐Earth3‐Veg_ssp245_2041–2060	28,300,710.7	2,912,680.9	17,704,649.4	2,929,606.1	10,655,268.8	0.0
EC‐Earth3‐Veg_ssp245_2061–2080	29,288,226.8	1,927,584.4	17,704,718.5	1,933,585.4	11,651,677.0	0.0
EC‐Earth3‐Veg_ssp585_2041–2060	29,325,808.0	1,888,925.4	17,704,718.5	1,897,014.5	11,689,285.6	0.0
EC‐Earth3‐Veg_ssp585_2061–2080	30,585,551.2	630,040.5	17,706,472.8	626,803.8	12,949,455.7	0.0

*Note:* Values are shown in hectares (ha). Circulation models (MPI‐ESM1‐2‐HR, EC‐Earth3‐Veg); scenarios of change (moderate ssp245 and critical ssp585) and projected over two time periods (2041–2060 and 2061–2080).
